# The Functionality Appreciation Scale (FAS): Psychometric properties and results of the Dutch version in a community sample and a sample of women with eating disorders

**DOI:** 10.1177/13591053251326002

**Published:** 2025-03-22

**Authors:** Marlies Rekkers, Aurélie Nieuwenhuijse, Mia Scheffers, Jooske T van Busschbach, Annemarie A van Elburg

**Affiliations:** 1 Utrecht University, The Netherlands; 2 Windesheim University of Applied Sciences, The Netherlands; 3 Emergis, The Netherlands; 4 University of Groningen, The Netherlands

**Keywords:** body functionality, body image, eating disorders, functionality appreciation, psychometric properties

## Abstract

Body satisfaction in eating disorders (EDs) is negatively affected by critical evaluation of aesthetic aspects. A focus on the appreciation for the functioning of the body could create a more positive perspective. The Functional Appreciation Scale (FAS) measures functional appreciation. This is the first study that examines the Dutch version of the FAS in a clinical sample. Psychometric properties and differences in scores are evaluated in an ED and a community sample consisting of women. Factor analysis identified and confirmed a one-dimensional factor structure. Reliability was adequate; convergent and discriminant validity were supported through correlations with the Body Cathexis Scale and the Eating Disorder Examination Questionnaire respectively. Women with EDs scored significantly lower than women from a community sample. Further research regarding the applicability of the FAS for clinical practice is needed because of the ceiling effect and the lack of strong invariance between the clinical and non-clinical group.

## Background

A negative body image or body dissatisfaction is recognized as a robust risk factor for the development, maintenance and relapse of eating disorders (EDs; Eshkevari et al., 2014; Rohde et al., 2015; Stice and Shaw, 2002). In people with EDs, body dissatisfaction predominantly focuses on negatively experienced aesthetic aspects of the body. However, body appreciation is not restricted to the way the body ‘looks’, but may also concern the way the body ‘functions’ (Abbott and Barber, 2010). Thus, the body has aesthetic qualities as well as functional qualities. According to Abbott and Barber (2011) these functional qualities concern perceptions of how the body feels, moves and functions. Both aesthetic and functional appreciation play a role in the level of body satisfaction.

Alleva et al. (2015) introduced the term body functionality for the functional aspects of body image. They describe body functionality as everything that the body does or is capable of doing and argue that it encompasses functions related to physical capacities, health and internal processes, bodily senses and perceptions, creative endeavours, communication with others and self-care. Body functionality appreciation is defined as ‘appreciating, respecting, and honouring the body for what it is capable of doing’ (Alleva et al., 2017, p.29). Body functionality appreciation could serve as an important aspect of positive body image and may be a useful target in the treatment of body dissatisfaction in EDs (Alleva et al., 2015; Rekkers et al., 2020; Walker and Murray, 2024). In their review on body functionality, Alleva and Tylka (2021) concluded that research underscores body functionality appreciation as a valuable construct with respect to positive body image and disordered eating. The authors state that appreciating body functionality rather than physical appearance may not only lead to more body satisfaction, but also to the ability to be more attuned to the body. For example, to be able to have an eating pattern based on listening to and following body cues such as hunger and satiety, instead of disordered eating cognitions. In this context, Baceviciene and Jankauskiene (2020) found that a functional body perception was a strong predictor of less disordered eating behaviour.

Studies in non-clinical groups, for example the study by Frisén and Holmqvist (2010), showed that when the evaluation of the body is more orientated towards function than appearance, it helps to increase positive body image. Also, Wood-Barcalow et al. (2010) concluded, based on a qualitative study among women studying at the university in the United States, that in women with a positive body image, a functional perception of the body is part of their body identity. Furthermore, in a sample of women studying psychology in the United Kingdom, Halliwell (2013) observed that functional aspects of body satisfaction may serve as a protective psychological mechanism against body dissatisfaction, contrary to aesthetic aspects. The above mentioned non-clinical studies show that a positive functional perception of the body or body parts may serve as an important ingredient of positive body image. However, little is known about the role of body functionality appreciation in the field of EDs.

Enhancing awareness of professionals and ED patients by measuring body functionality appreciation in a clinical context, may lead to more diagnostic information and to a better insight into the course and effect of the treatment of a negative body image. Abbott and Barber (2010) considered it a limitation that most body image questionnaires fail to incorporate the functional dimension. They found that especially women do not evaluate or reflect spontaneously on functional aspects of their body. A questionnaire which incorporates body functionality may clarify and sensitize these aspects during a diagnostic phase. Furthermore, measuring body functionality appreciation creates the possibility to monitor the effectiveness of therapeutic interventions which focus on functional aspects of body image to enhance body satisfaction. In this light, Rekkers et al. (2020, 2022) developed and evaluated a protocol based on *positive* body exposure in which functionality appreciation forms an important and innovative part. Recently, Walker and Murray (2024) developed a novel body functionality-focussed mirror exposure, integrating a complete focus on body functionality with mirror exposure.

In order to gain a better understanding of the efficacy of these interventions, questionnaires are needed to evaluate the role and influence of body functionality appreciation as a dimension of positive body image. In the last decades two questionnaires have been presented, which measure functionality appreciation besides aesthetic appreciation of the body. In 2010, Abbott and Barber (2010) developed the Embodied Image Scale (EIS), which measures the cognitive, behavioural and affective components of body image across the functional and aesthetic dimensions of body image. In a sample of Australian adolescents, the EIS demonstrated acceptable validity and reliability. Recently, Rekkers et al. (2021) described functional body appreciation as a separate factor in the Body Cathexis Scale (BCS; Secord and Jourard, 1953) in both a non-clinical sample and a clinical ED sample. The BCS measures the degree of appreciation for both the appearance and functioning of different parts of the body and has been proven a valid and reliable questionnaire in various studies (Orlandi et al., 2006; Scheffers et al., 2019). Although these two questionnaires are available, a questionnaire assessing the construct of body functionality appreciation separately was missing. For this reason, Alleva et al. (2017) developed the Functionality Appreciation Scale (FAS).

The FAS is a 7-item questionnaire measuring body functionality appreciation. Alleva et al. (2017) examined its psychometric properties among three online adult community samples of women and men in the U.S. They found that FAS-scores demonstrated criterion-related and construct validity and were internally consistent and stable across a 3-week period. Exploratory and confirmatory factor analysis revealed a unidimensional structure. Shortly thereafter, more psychometric studies on the FAS were conducted in Malaysia (Swami et al., 2019), Australia (Linardon et al., 2020), Brazil (Faria et al., 2020), Italy (Cerea et al., 2021), Romania (Swami et al., 2021), Japan (Namatame et al., 2022), Lebanon (Swami et al., 2022), Greece (Anastasiades et al., 2023), the Netherlands (Alleva et al., 2023) and Spain (Zamora et al., 2024). Despite this considerable number of studies, the FAS has never been examined in a clinical ED sample with comparisons made to a matching community sample. Therefore, it is unclear to what extent the instrument’s psychometric properties will be upheld in a sample consisting of patients with EDs. Furthermore, it is relevant to explore differences in FAS-scores between non-clinical and clinical samples.

To expand the existing knowledge on the FAS, the first goal of the present study is to evaluate the psychometric properties (factor structure, construct validity and reliability) of the FAS in a Dutch clinical sample of women with ED and compare these with those shown in an equivalent community sample. We expect, based on prior research, that the Dutch translation (Alleva et al., 2023) will demonstrate a one-dimensional factor structure in both exploratory and confirmatory factor analyses. Evidence of convergent construct validity will be established through positive associations with body appreciation, measured with the BCS (Rekkers et al., 2021), especially with the subscale Functionality of the BCS. We will also examine negative associations with the subscales weight concern and shape concern of the Eating Disorder Examination Questionnaire (EDE-Q; Fairburn and Beglin, 1994). Furthermore, we will examine discriminant construct validity. Taking in consideration the finding by Baceviciene and Jankauskiene (2020) that body functionality was a strong predictor of less disordered eating behaviour, we hypothesize that FAS scores will correlate negatively with the total score of the EDE-Q, in particular with the subscales restraint and eating concern. Finally, we will assess test-retest reliability of FAS scores over a 2-week interval.

The second goal is to explore differences in body functionality appreciation measured with the FAS between men and women in the community sample and differences in body functionality appreciation between women with EDs and women in the community sample. Alleva et al. (2023) already demonstrated gender invariance in Dutch FAS scores in a community sample, which enables us to compare levels of functionality appreciation between women and men. Based on the results of their study we also expect to find significant gender differences in our study. In line with the study by Rekkers et al. (2021) in which data on the functionality scale of the BCS was used, it is hypothesized that the FAS will reveal a significantly lower body functionality appreciation in people with EDs compared to respondents from a community sample.

## Method

### Participants

Two independent samples were used in this study. The ED sample was recruited in 13 specialized centres for ED across the Netherlands between February 2021 and November 2021 and consisted of 156 participants with EDs. Inclusion criteria for participation were currently in treatment in one of the centres and age older than 18. Only a very small minority of participants were male (women *n* = 150, men *n* = 5, unknown *n* = 1). For this reason, we limited our ED sample to women in order to create a gender homogeneous sample. The 150 women were diagnosed according to DSM-5 criteria in the following categories: Anorexia Nervosa (AN) 62.7% (*n* = 94), Bulimia Nervosa (BN) 9.3% (*n* = 14), Binge Eating Disorder (BED) 13.2% (*n* = 20), Otherwise Specified Feeding and Eating Disorder (OSFED)14% (*n* = 21) and Avoidance Restrictive Food Intake Disorder (ARFID) 0.7% (*n* = 1). Mean age was 29.45 (*SD* 10.44, range 18–67) and self-reported mean body mass index (BMI) was 22.13 (*SD* 7.62, range 12.70–48.65). Ninety-five women followed an outpatient ED treatment and 55 an inpatient ED treatment, with an average treatment duration of 8.9 months (*SD* = 11.51, range 1–96).

The community sample consisted of 982 individuals from the general Dutch population, recruited between November 2019 and May 2020. The inclusion criteria were age older than 18 years and absence of ED symptoms, according to scores on the EDE-Q. Following these inclusion criteria, we excluded participants younger than 18 (*n* = 17) from the original sample and also removed participants with an EDE-Q score more than two standard deviations above the mean (*n* = 40). We did not exclude participants with a very high or very low BMI, when their EDE-Q scores gave no indication for ED symptoms. The sample of the remaining 925 participants consisted of 669 women and 256 men with an average age of 36 years (*M* = 36.53, SD = 14.71, range 18–76) and an average self-reported BMI of 23.97 (*SD* = 3.64, range 13.93–41.10). To be able to compare the community sample and the ED sample we excluded the men in the community sample in our psychometric analyses and used a sample of participants of only women with an average age of 36.03 (*SD* = 14.10, range 18–76) and an average self-reported BMI of 23.71 (*SD* = 3.83, range 13.93–41.10).

### Measures

The FAS (Alleva et al., 2017; Dutch version: Alleva et al., 2023) contains seven items rated on a 5-point Likert scale (1 = ‘strongly disagree’ to 5 = ‘strongly agree’). Scores from all items are averaged to produce a total score, with higher scores reflecting greater functionality appreciation. Internal consistency of the Dutch version was good with Cronbach’s alpha 0.91 for the total sample and a test-retest reliability (ICC) of 0.72 (Alleva et al., 2023).

The BCS (Secord and Jourard, 1953; Dutch version: Dorhout et al., 2015) measures the degree of appreciation for the appearance and functioning of different parts of the body. The BCS consists of 40 items rated on a 5-point Likert scale (1 = ‘very dissatisfied’ to 5 = ‘very satisfied’). Higher scores indicate a higher body satisfaction. The construct and concurrent validity of the original scale are good (Orlandi et al., 2006; Ward, 1994). Research on the Dutch version of the BCS in both an ED (*n* = 238) and a community (*n* = 1060) sample revealed three subscales: functional body satisfaction, weight related body satisfaction and non-weight related body satisfaction (Rekkers et al., 2021). Internal consistency was good in both samples with Cronbach’s α = 0.90 for the total scale and Cronbach’s α = 0.83 − .85 for the subscales in the clinical sample (Rekkers et al., 2021).

The EDE-Q (Fairburn and Beglin, 1994; Dutch version: Aardoom et al., 2012) measures ED symptoms. The EDE-Q consists of 36 items of which 22 determine the total score. These 22 items comprise four subscales, assessing restraint, shape concerns, weight concerns and eating concerns over the previous 28 days. Items are answered on a 7-point Likert scale ranging from 0, ‘not one day’, to 6, ‘every day’. Higher scores are indicative of higher ED psychopathology. Internal consistency of the Dutch version is good, with Cronbach’s α = 0.95 for the total scale and varying from 0.81 to 0.91 for the subscales (Aardoom et al., 2012).

### Procedure

The study protocol was approved by the ethics committee of the Faculty of Social and Behavioural Sciences of Utrecht University (number 20–383). Furthermore, the Medical Ethics Review Committee of Utrecht University was consulted on this procedure; reference number WAG/mb/20/022653.

To collect clinical data the project, that also included questionnaires on physical activity, was advertised in the 13 centres for ED as a study on ‘Measuring exercise behaviour and body image in people with EDs’, by means of a poster and accompanying flyers including a QR-code. To participate in the study, ED patients could scan the QR-code on the flyer. After providing digital informed consent, information about the study objective and the voluntary and anonymous participation, participants were asked to complete an online survey via Qualtrics (www.qualtrics.com).

Data collection for the community sample was conducted using a snowball sampling method through e-mails sent to potential participants in the network of six bachelor students from Windesheim University of Applied Sciences, School of Human Movement and Sports and the network of the first two researchers of this study. The e-mail included a link to the questionnaires preceded by an informed consent, information about the study objective and the voluntary and anonymous participation, and a request to readers to forward the e-mail to others in their network. Participants completed the survey through a secure online system (Formdesk) and could indicate if they were willing to fill out the survey for a second time. If this was the case, they got a personal code and received the survey again after 2 weeks. One hundred sixteen participants, all women, completed the survey twice within a 3-week interval. All survey materials were removed from the internet upon completion of the data collection phase.

Informed consent was obtained from all individual participants included in this study, both clinical and non-clinical. In both samples no participatory incentives were offered.

### Statistical analysis

The factor structure of the Dutch FAS was examined using a two-step procedure that involved exploratory factor analysis (EFA) in the first step and confirmatory factor analysis (CFA) in the second step (Swami and Barron, 2019). This procedure was followed since this was the first time that the factor structure of the FAS in an ED-sample was tested using EFA and CFA and it is unclear to what extent the instrument’s psychometric properties will be upheld in such a clinical sample. The ED sample consisting of only women was therefore split, using a computer-generated random seed, and, for reasons of comparison, the same procedure was followed for the participants of the community sample that also included only women. For the EFA this resulted in a split-half sample of women with ED (*n* = 75), and a split-half community sample of women (*n* = 331). For the CFA the following split-half samples were used: a sample of women with ED (*n* = 75) and a community sample of women (*n* = 338). For the EFA, maximum likelihood was used as the factor extraction method (Costello and Osborne, 2005) using SPSS 28.0. Numbers of factors retained were based on interpretation of the scree plot (Watkins, 2018) and parallel analysis (Hayton et al., 2004). Cross-loadings were defined as an item that loads at > 0.32 on two or more factors (Tabachnick and Fidell, 2014). For the CFA, maximum likelihood with robust standard errors (MLR) was used and fit indices were interpreted applying the Satorra-Bentler correction (Satorra and Bentler, 2001). We chose to report a broad range of indices and included root mean square error of approximation (RMSEA), standardized root mean square residual (SRMR), Comparative Fit Index (CFI) and Tucker Lewis Index (TLI). As a rule of thumb, RMSEA values < 0.08 suggest adequate and <.05 good model fit (Browne and Cudeck, 1993). An SRMR between 0.05 and 0.10 indicates an acceptable fit and values <.05 indicate good fit (Schermelleh-Engel et al., 2003). CFI and TLI values in the range between 0.90 and 0.95 may be regarded as indicating good model fit (Brown, 2015).

We performed multiple group analyses to investigate measurement invariance across the clinical and community sample. Invariance is a prerequisite for group comparisons to reflect true differences (Brown, 2015; Rusticus et al., 2008), not due to systematic differences in interpretation of items due to respondents’ group membership. The extent of measurement invariance was evaluated in a series of three models. In model A, specifying ‘configural invariance’, the same factor structure is imposed on the two groups. In the next model (B), specifying ‘weak invariance’, the factor loadings are constrained to be equal across groups. In Model C, ‘strong invariance’, the factor loadings *and* intercepts are constrained to be equal across groups. The model selection was performed by testing invariance by the Scaled Difference in Chi-Squares (SDCS) test (Satorra and Bentler, 1994) for nested models estimated with MLR. Inspection of size and consistency of factor loadings were performed to further evaluate model fit (Brown, 2015). This sequential model estimation procedure to study measurement invariance is used since lack of strong factorial invariance will contaminate estimates of group mean differences (Rusticus et al., 2008). It is widely acknowledged however, that the requirement of strong factorial invariance may be too strict and unrealistic a goal for group comparisons. Consequently, Byrne et al. (1989) introduced the concept of partial invariance in which only a subset of parameters in each subscale must be invariant whereas others are allowed to vary between the groups. Partial invariance was investigated by inspecting modification indices to determine which cross-group equality constraint most significantly contributed to lack of fit; the model was re-estimated after freeing that constraint. Partial invariance of certain items signals qualitative group differences that render exact between-group comparisons including these items possibly less meaningful.

To examine construct and discriminant validity, correlations between functional appreciation, body appreciation in general and ED pathology were analysed using Pearson product-moment correlation coefficient with correlations considered strong if *r* = 0.50–1.0, moderate if *r* = 0.30 − .49 and weak if *r* = 0.10–0.29 (Cohen, 1988). To assess the internal consistency for the FAS items, we calculated Cronbach’s alpha and, for completeness, also McDonald’s ω using the so-called HA algorithm that estimates item loadings based on products, ratios, and sums of the covariances of item responses. This algorithm, integrated in the OMEGA Macro by Hayes, also provides for confidence intervals for ω (Hayes and Coutts, 2020). Whereas Cronbach’s alpha assumes equal factor, ω can be used as an estimator of reliability when this condition is not met. McDonald’s ω values above 0.70 are considered adequate (Dunn et al., 2014). Test-retest reliability of the FAS scores was established by intraclass correlation (ICC; two-way mixed model, absolute agreement, single measurement; Perinetti, 2018). ICC ≥ 0.75 was considered excellent, between 0.60 and 0.74 good, between 0.40 and 0.59 fair and below 0.40 poor (Cicchetti, 1994).

An independent t-test was used to analyse possible differences in FAS scores between men and women in the community sample, a comparison made under the assumption of gender invariance that was found earlier in a Dutch community sample (Alleva et al., 2023) and in other Western community samples (Alleva et al., 2017; Cerea et al., 2021; Zamora et al., 2024). Differences in FAS scores in the community and ED sample of women were also explored with an independent t-test.

## Results

### Exploratory factor analysis

The Kaiser-Meyer-Olkin (KMO) scale verified the sampling adequacy for the EFA on the community sample, with KMO = 0.887 (‘good’, according to Field (2009); Barlett’s test of sphericity was statistically significant (χ^2^ = 1039.25; df = 21, *p* < 0.0001), indicating that data were suitable for exploratory factor analysis. Inspection of the scree plot was employed to determine the appropriate number of factors to retain. The scree plot showed an inflexion justifying one factor. Also, parallel analysis (O’connor, 2000) suggested that the seven FAS items converged into one factor, accounting for 58.34% of common variance (see Table 1). In the EFA on the ED sample KMO was 0.897 (‘good’ according to Field (2009) and Barlett’s test of sphericity was statistically significant (χ^2^ = 336.92; df = 21, *p* < 0.0001). Both the scree plot and parallel analysis justified one factor, accounting for 67.18% of the total item variance (see Table 1).

**Table 1.** Items of the functionality appreciation scale in English and Dutch (in italics) and factor loadings derived from exploratory factor analysis with the community sample and the ED sample consisting of women.

Item	Community sample (*n* = 331)	ED sample (*n* = 75)
(1) I appreciate my body for what it is capable of doing / *Ik waardeer mijn lichaam voor wat het in staat is om te doen*	0.738	0.804
(2) I am grateful for the health of my body, even if it isn’t always as healthy as I would like it to be / *Ik ben dankbaar voor de gezondheid van mijn lichaam, zelfs als het niet altijd zo gezond is als ik graag zou willen*	0.731	0.799
(3) I appreciate that my body allows me to communicate and interact with others / *Ik waardeer dat mijn lichaam me de mogelijkheid geeft om te communiceren en in contact te zijn met anderen*	0.657	0.777
(4) I acknowledge and appreciate when my body feels good and/or relaxed/ *Ik erken en waardeer het wanneer mijn lichaam goed en/of relaxt voelt*	0.605	0.627
(5) I am grateful that my body enables me to engage in activities that I enjoy or find important/ *Ik ben dankbaar dat mijn lichaam me in staat stelt om deel te nemen aan activiteiten die ik leuk of belangrijk vind*	0.780	0.866
(6) I feel that my body does so much for me*/ Ik vind dat mijn lichaam heel veel voor mij doet*	0.736	0.799
(7) I respect my body for the functions it performs / *Ik respecteer mijn lichaam voor de functies die het uitvoert*	0.763	0.817

### Confirmatory factor analysis

CFA showed a good fit for the one-factor model that resulted from the EFA in the community sample. In the clinical sample the fit was also good according to SRMR, CFI and TFI, although RMSEA indicated that fit was not fully adequate (see Table 2).

**Table 2.** CFA of the community sample of women (*n* = 338) and the ED sample of women (*n* = 75).

Factor	Sample	*χ* ^2^	*df*	*RMSEA (90% CI)*	*SRMR*	*CFI*	*TLI*
1	ED	220.070	21	0.000 (0.000 − .077)	0.037	1.000	1.045
1	Community	1172.472	21	0.090 (0.065 − .116)	0.040	0.948	0.922

*χ*^2^ = chi square; *df* = degrees of freedom; *RMSEA* = root mean square error of approximation 90% CI = 90% confidence interval of the RMSEA; *SRMR* =standardized root mean square residual; *CFI* =comparative fit index; TLI = Tucker Lewis index.

### Reliability

Cronbach’s alpha’s for the FAS in the community sample of women and ED sample of women were 0.88 and 0.90 respectively. McDonald’s ω was equal to these alpha’s, that is 0.88 (95% CI = 0.86 to 0.90) in the community sample and 0.90 for the ED sample (95% CI = 0.86 to 0.92). The intraclass correlation coefficient (ICC) between test and retest FAS-scores in the community sample of women was 0.70.

### Invariance across groups

A multiple-group confirmatory factor analysis showed weak invariance, but did not support strong invariance across the ED sample and the community sample. Item four was identified as contributing most to the lack of strong invariance across groups (see Table 3). We examined the differences in averages between a FAS version with or without item four. In the ED sample there was a slight difference between FAS 7-items (*M* = 3.29; *SD* = 0.58) and FAS 6-items (*M* = 3.33; *SD* = 0.86), in the community sample the averages were the same (*M* = 4.26 with respectively *SD* = 0.57 and 0.59; see Table 4). We decided to retain all seven items because the net impact of the differences on the total mean scores is small.

**Table 3.** Measurement invariance across the community sample and the ED sample.

Model		*χ* ^2^	*df*	scl	Δ*χ*^2^	Δ*df*	T_s_	RMSEA (90% CI)	SRMR	CFI	TLI
1A	configural invariance	65.890	28	1.3401				0.081 (0.056 −0.106)	0.040	0.958	0.937
1B	weak invariance	71.389	34	1.3243	5.499	6	4.70	0.073 (0.049–0.097)	0.063	0.959	0.949
1 C	strong invariance	95.315	40	1.2738	23.926	6	21.55*	0.082 (0.061 −0.103)	0.095	0.958	0.937
1 C-1	partial strong item d4^a^	83.271	38	1.287	11.882	4	10.80*	0.076 (0.0540 −0.098)	0.082	0.950	0.945

χ^2^ = chi square; df = degrees of freedom; T_s_ = Scaled Difference in Chi-Squares (SDCS) test statistic; RMSEA = Root Mean Square Error of Approximation; 90% CI = 90%; confidence interval of the RMSEA; SRMR = Standardized Root Mean Square Residual; CFI = comparative fit index; TLI = Tucker Lewis index. scl = scaling correction factor, *p < .05.

a modification index item d4 = 10.201.

**Table 4.** The mean of all items of the FAS.

ED Sample			Community sample	
item	*M*	*SD*	*M*	*SD*
1	3.11	1.03	4.14	0.75
2	3.33	1.03	4.21	0.73
3	3.52	1.03	4.32	0.75
4	3.05	1.19	4.28	0.74
5	3.59	1.05	4.42	0.72
6	3.24	1.08	4.17	0.80
7	3.19	1.10	4.28	0.71

*M* = Mean, SD = Standard Deviation.

### Preliminary analyses

In the community sample, no significant difference between FAS-scores of men and women was found (*t* (923) = −0.555; *p* = 0.479). FAS-scores of women in the community and the ED sample differed significantly (*t* (817) = 17.103; *p* = 0.0001). For means and *SD* of all questionnaires of both samples see Table 5.

**Table 5.** Means and SD of all questionnaires in the community sample (total, men and women) and in the ED sample of women.

Measures	Non-clinical Total (*n* = 925)	Non-clinical Men (*n* = 256)	Non-clinical Women (*n* = 669)	Clinical Women (*n* = 150)
	*M (SD)*	*M (SD)*	*M (SD)*	*M (SD)*
FAS BCS Total	4.24 (0.57) 3.62 (0.52)	4.24 (0.57) 3.77 (0.51)	4.26 (0.57) 3.57 (0.51)	3.29 (0.85) 2.73 (0.52)
-BCS-NW	3.71 (0.54)	3.81 (0.55)	3.68 (0.53)	2.94 (0.53)
-BCS-W	3.44 (0.78)	3.73 (0.64)	3.33 (0.79)	1.90 (0.76)
-BCS-F	3.57 (0.60)	3.72 (0.57)	3.52 (0.61)	2.85 (0.61)
EDE-Q Total †	1.07 (0.87)	0.86 (0.76)	1.15 (0.90)	3.78 (1.11)
-EDE-Q-R	1.10 (1.10)	1.08 (1.14)	1.12 (1.08)	3.38 (1.41)
-EDE-Q-EC	0.43 (0.66)	0.36 (1.14)	0.45 (1.08)	2.92 (1.13)
-EDE-Q-WC	1.33 (1.16)	0.96 (0.94)	1.48 (1.20)	4.16 (1.29)
-EDE-Q-SC	1.42 (1.19)	1.05 (0.95)	1.57 (1.24)	4.65 (1.17)

*M* = mean, *SD* = standard deviation, FAS = Functionality Appreciation Scale, BCS = Body Cathexis Scale, BCS-NW = Body Cathexis Scale-Non-Weight, BCS-W = Body Cathexis Scale-Weight, BCS-F = Body Cathexis Scale-Functionality, EDE-Q = Eating Disorder Examination Questionnaire, EDE-Q-R = Eating Disorder Examination Questionnaire-Restraint, EDE-Q-EC = Eating Disorder Examination Questionnaire-Eating Concern, EDE-Q-WC = Eating Disorder Examination Questionnaire Weight Concern. EDE-Q-SC: Eating Disorder Examination Questionnaire Shape Concern, † For EDE-Q results ED sample of women: *n* = 142.

### Convergent and discriminant construct validity

Pearson’s correlations between the FAS and the BCS total score and subscales were positively correlated in the community sample and the ED sample, with correlations varying from moderate to strong. Pearson’s correlations between the FAS and the EDE-Q total score and subscales were negatively correlated in the ED sample varying from weak (EDE-Q-R), moderate (EDE-Q-EC & EDE-Q-WC) to strong (EDE-Q-SC). In the community sample these correlations are all weak. (see Table 6).

**Table 6.** Pearson correlations between FAS and EDE Q and BCS scores in the ED sample (*n* = 166) and the Community sample (*n* = 669).

	FAS	
Measures	ED sample	Community sample
EDE-Q total	−0.454**	−0.274**
EDE-Q-R	−0.263**	−0.108**
EDE-Q-EC	−0.374**	−0.238**
EDE-Q-WC	−0.480**	−0.283**
EDE-Q-SC	−0.510**	−0.297**
BCS total	0.589**	0.510**
BCS-NW	0.556**	0.420**
BCS-W	0.475**	0.400**
BCS-F	0.476**	0.468**

FAS = Functionality Appreciation Scale, EDE-Q = Eating Disorder Examination Questionnaire, EDE-Q-R = Eating Disorder Examination Questionnaire Restrictive, EDE-Q-EC = Eating Disorder Examination Questionnaire Eating Concern, EDE-Q-WC = Eating Disorder Examination Questionnaire Weight Concern, EDE-Q-SC = Eating Disorder Examination, **p < .001.

## Discussion

In this study we examined the psychometric properties of the FAS in an ED sample and a community sample, both consisting of women. We also explored differences between these two samples in body functionality appreciation as measured with the FAS and between men and women in the whole community sample. In terms of the dimensionality of the FAS, EFA identified a one-dimensional factor structure, which was confirmed by CFA, revealing adequate fit values for a one-factor model in both the ED sample of women and the community sample of women. These results are in line with previous studies on the FAS in community samples. Internal consistency was good in both samples. Test-retest reliability in the community sample, consisting of women, was good.

A multiple-group confirmatory factor analysis showed weak invariance, but did not support strong invariance across the ED sample and the community sample. Item four was identified as contributing most to the lack of strong invariance across groups. This result corresponds with those of Todd and Swami (2020) that supported partial strong invariance across nations, after fixing the intercept for item four. Millsap and Kwok (2004) describe the option of omitting the items that performed differently across groups, at the same time warning that this option results in as many (shortened) versions of a scale as there are invariance studies. A second option is to retain all items when the net impact of the differences on the total score is small (Millsap and Kwok, 2004) as is the case in our study. Next to the technical identification of this differing item, there are arguments from a qualitative perspective as to why item four might differ across groups. Looking closer, item four (‘I acknowledge and appreciate when my body feels good and/or relaxed’) could lead to different interpretations. One can acknowledge something, but not appreciate it; and 1’s body can feel good, but this doesn’t necessarily mean that it feels relaxed. The formulation of this item creates different possible perspectives dependent on which part of the item one responds to, which could have more impact in different cultural or clinical groups.

Strong positive correlations were found between the FAS and the total BCS-scores in the ED sample and the community sample, supporting the convergent validity of the Dutch FAS in these two samples. Contrary to our expectations, the subscale Functionality of the BCS did not have a stronger positive correlation with the FAS scores than the two other subscales Weight and Non-Weight of the BCS in both samples. The explanation could be that the FAS and the subscale Functionality of the BCS do not entirely measure the same construct of functionality appreciation. We already mentioned that Alleva et al. (2017, p. 29) designed the FAS to measure functionality appreciation as “appreciating, respecting, and honouring the body for what it is capable of doing” whereas the items on the BCS subscale Functionality only ask for 1’s satisfaction about functional aspects of body parts (body processes). In addition, all the items of the FAS refer to the functioning of the body as a whole, whereas the items of the BCS refer to specific body parts or body processes. In this respect it is important to note that clinical observations show that, when ED patients speak or think about their body, they only tend to make connection with the middle part of their body, the part they often disgust and objectify. Using the term body in the FAS-items instead of naming different body parts could unintentionally stimulate negative thoughts and feelings when specifically, ED patients only visualize their torso as an equivalent of their body. For this reason, the answers on the FAS could contrast with those on specific body parts or body processes such as the BCS items, which in turn encourage perception and awareness that 1’s own body is more than a disliked torso. We recommend to use both the BCS and the FAS in the field of EDs in order to get a more complete and more accurate picture of body functionality.

The subscales weight concern and shape concern were, as expected, negatively correlated with the FAS. It is remarkable that in the ED sample, these correlations are strong (shape concern) and moderate (weight concern), while in the community sample these correlations are both weak. It is possible that, when body image concern is part of an ED, it has a stronger negative association with functionality appreciation. As hypothesized, the EDE-Q total and the subscales eating restraint and eating concern in the ED sample were subsequently moderate, weak and moderate negatively correlated with FAS scores. These outcomes differ from those in our community sample of women (only weak associations), but also from the results in the study by Alleva et al. (2017) in a U.S. online community sample of women and men, where the negative correlations were even lower and not significant (eating restraint: *r* = 0.04; eating concern: *r* = −0.13) The negative correlations in our ED sample indicate that in women with a diagnosis of ED lower scores on body functionality appreciation are associated with higher scores of the severity of the ED. This finding may have clinical relevance, because it suggests that in women with ED functionality appreciation may possibly attenuate ED-symptoms. This is in line with Linardon et al. (2023) who state that body functionality appreciation may even promote adaptive eating patterns.

Consistent with the findings from previous studies (Alleva et al., 2017; Swami et al., 2021; Zamora et al., 2024) we found no significant gender difference in FAS scores in our Dutch community sample. It is remarkable that in a recent study of Alleva et al. (2023), which also used a Dutch community sample, women (*n* = 255) reported significantly higher functionality appreciation compared to men (*n* = 204). The authors speculate that gender differences in FAS scores may be the exception, rather than the norm. With our findings indeed confirming this pattern, the question remains as to the causes of the variation in results.

Importantly and confirm hypothesis, there was a significant difference between FAS scores in the ED sample and the community sample. This suggests that women with EDs are not only less satisfied with their appearance but, although to a lesser extent, also have a lower appreciation of how their body functions than women in a community sample. This difference and the ability to accurately measure this difference with the FAS are important when considering its clinical application in the field of EDs. However, because of the lack of strong invariance between the clinical and non-clinical group, comparisons between the two groups should be interpretated with caution.

The present study has two major limitations and a point to consider. A significant limitation concerns the low number of male respondents in the original ED sample. In this study the community sample (*n* = 925) consisted of an acceptable balanced mix of adult men and women of different ages, without ED symptoms, which gives indications of community norm scores for women as well as for men of the Dutch version of the FAS. However, only five men participated in the clinical sample in this study. Another limitation in our study was the disproportionate distribution of the various types of EDs in our clinical sample. The majority of them were women with AN (62.7%) and the percentages of women with BN (9.3%) and ARFID (0.7%) were too low to make valid comparisons

A point to consider is that mean FAS scores were high and in the community sample even much higher than the scale mid-point (men: *M* = 4.26; women: *M* = 4.24), suggesting, as also argued by Swami et al. (2019) and Zamora et al. (2024), a ceiling effect. Swami et al. (2019) explain these high means by stating that it may be difficult for individuals to disagree with FAS items, because they are formulated to be generally applicable. Another observation in the present study, which could also create a ceiling effect, is that FAS-items do not seem sufficiently distinctive from each other, with closely spaced item loadings between 0.605 and 0.780 (community sample) and 0.627 and 0.866 (ED sample). The ceiling effect possibly also accounts for the near perfect model fit found in the CFA. In summary, we conclude that this ceiling effect could limit the use of the FAS in research settings, as comparisons between clinical and non-clinical groups become more difficult, and may be inaccurate. Alleva et al. (2017) also warned about the ceiling effect in the context of programmes that aim to enhance functionality appreciation.

Considering the limitations of our study, future research should include a relevant clinical sample of appropriate size to address possible differences in body functionality appreciation within the ED population, for example, between men and women with EDs but also between people with different sub diagnoses. A second recommendation would be to gather more data on variations of FAS scores over time both in community and clinical samples. Given that therapeutic interventions have been developed in the field of EDs, which also focus on (Rekkers et al., 2020; Tanck et al., 2021) or entirely address (Walker and Murray, 2024) functional aspects of body image to enhance body satisfaction, we agree with Yurtsever et al. (2022) that it is essential to explore the sensitivity to change of the FAS and if justified by the data, develop reliable change indices and possibly a measure of relevant clinical change.

### Conclusions

This study of the FAS provides evidence that the Dutch version of the FAS is one-dimensional, has a good reliability as well as adequate construct validity in both women with and without ED. The FAS scores showed that women with EDs have a lower level of body appreciation concerning body functionality compared to healthy women although this distinction is not as large as in the case of appearance related body appreciation. Within the group women with an ED, those with a more positive view on the way their body functions, report a less severe disordered eating behaviour pattern. This study demonstrates satisfactory psychometric results of the Dutch version of the FAS, but also shows that further research regarding the applicability of the FAS for clinical practice is needed because of the ceiling effect and the lack of strong invariance between the clinical and non-clinical group.
